# A systematic approach to analyze the social determinants of cardiovascular disease

**DOI:** 10.1371/journal.pone.0190960

**Published:** 2018-01-25

**Authors:** Mireya Martínez-García, Magaly Salinas-Ortega, Iván Estrada-Arriaga, Enrique Hernández-Lemus, Rodrigo García-Herrera, Maite Vallejo

**Affiliations:** 1 Sociomedical Research Department, National Institute of Cardiology, Mexico City, Mexico; 2 Metropolitan Autonomous University (UAM), Xochimilco, Mexico City, Mexico; 3 Health Science School, University of the Valley of Mexico (UVM), Mexico City, Mexico; 4 Computational Genomics Division, National Institute of Genomic Medicine, Mexico City, Mexico; 5 Center for Complexity Sciences, National Autonomous University of Mexico, Mexico City, Mexico; The Chinese University of Hong Kong, HONG KONG

## Abstract

Cardiovascular diseases are the leading cause of human mortality worldwide. Among the many factors associated with the etiology, incidence, and evolution of such diseases; social and environmental issues constitute an important and often overlooked component. Understanding to a greater extent the scope to which such social determinants of cardiovascular diseases (SDCVD) occur as well as the connections among them would be useful for public health policy making. Here, we will explore the historical trends and associations among the main SDCVD in the published literature. Our aim will be finding meaningful relations among those that will help us to have an integrated view on this complex phenomenon by providing historical context and a relational framework. To uncover such relations, we used a data mining approach to the current literature, followed by network analysis of the interrelationships discovered. To this end, we systematically mined the PubMed/MEDLINE database for references of published studies on the subject, as outlined by the World Health Organization’s framework on social determinants of health. The analyzed structured corpus consisted in circa 1190 articles categorized by means of the *Medical Subheadings* (MeSH) content-descriptor. The use of data analytics techniques allowed us to find a number of non-trivial connections among SDCVDs. Such relations may be relevant to get a deeper understanding of the social and environmental issues associated with cardiovascular disease and are often overlooked by traditional literature survey approaches, such as systematic reviews and meta-analyses.

## Introduction

According to estimates from the World Health Organization (WHO) in 2015, 17.7 million people died from CVD, representing a 31% of all deaths due to non-communicable diseases of which 7.4 million were related to ischemic heart disease (IHD) and 6.7% for stroke [1].

Several health conditions and risk factors are associated with the incidence of cardiovascular diseases (CVD) such as: hypertension, diabetes mellitus, tobacco smoking, dyslipidaemia, unhealthy alcohol consumption and physical inactivity, for which preventive interventions, towards the reduction of population exposure to behavioural risk factors and early detection and treatment of people who are already expose, have been taken. However, countries have been impacted differently because these approaches depend on multi-sectoral consultation and multi-stakeholder collaboration, for which low and middle income countries have limited addressing capacity.

Global disease burden has changed over the last two decades shifting from communicable to non-communicable diseases, being CVDs the largest contributor worldwide, accounted 393.8 million (14.4%) of total global disability-adjusted life-years (DALYs) lost in 2012; this epidemiological transition is closely related to the socio-economic development of each country among other factors.

As for the economic consequences related to non-communicable disease, there is a combined effect between health care costs and lost of economic productivity due to illness and premature deaths imposing catastrophic expenditure among the uninsured population [2].

Social, environmental and economic factors may play an important role in the development, evolution and outcomes of cardiovascular diseases among populations. For that reason, the WHO created in 2005 the Commission on Social Determinants of Health (CSDH) to promote health equity between and within countries by focusing on the causes of the causes [3].

In this context the WHO defines the social determinants of health (SDH) as *… the circumstances in which people are born, grow, live, work, and age, and the systems put in place to deal with illness…* [4], thus highlighting that health and ilnesses are not distributed homogeneously throughout human societies (due to disparities in these SDHs) and neither are the resources to deal with health issues.

**Fig 1:**
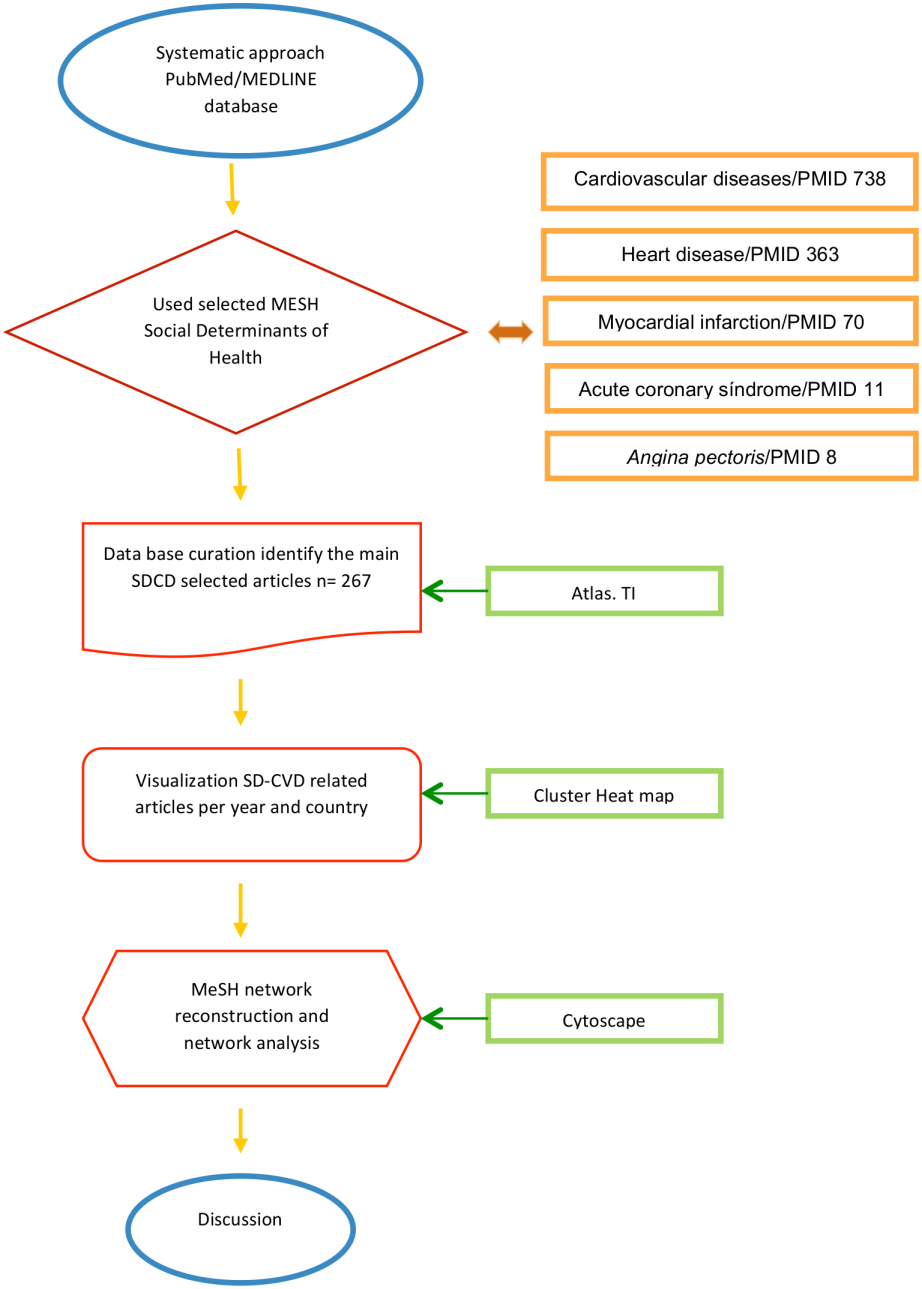
Workflow for the analytical research performed. Once a systematic PubMed search procedure has been performed with the *Social Determinants of Health* MeSH as main parameter and cardiovascular disease terms as modifiers, a series of data curation procedures was made by using e-QAD tool *Atlas.TI*. The curated corpus was used to obtain data visualizations and to infer semantic-like MeSH Networks [13]. MeSH networks were later analyzed to look up for non-trivial associations. Network and visualization results were then discussed.

The CSDH has proposed a conceptual framework (CSDH-FW) to guide empirical work to enhance our understanding of the complexity of social determinants of health within a public health context [5]. This consists of public policies, scientific traditions and multiple other features that affect population health. The ultimate goal of such CSDH-FW is to highlight the different levels of causation (such as sociopolitical context) that define the structural mechanisms to generate social stratification, class divisions and socioeconomic position, in order to account for them in the design of public health policy [6]. The present study (as outlined in Fig 1, that will be explained later) is an attempt to an unbiased analysis of these determinants.

**Fig 2:**
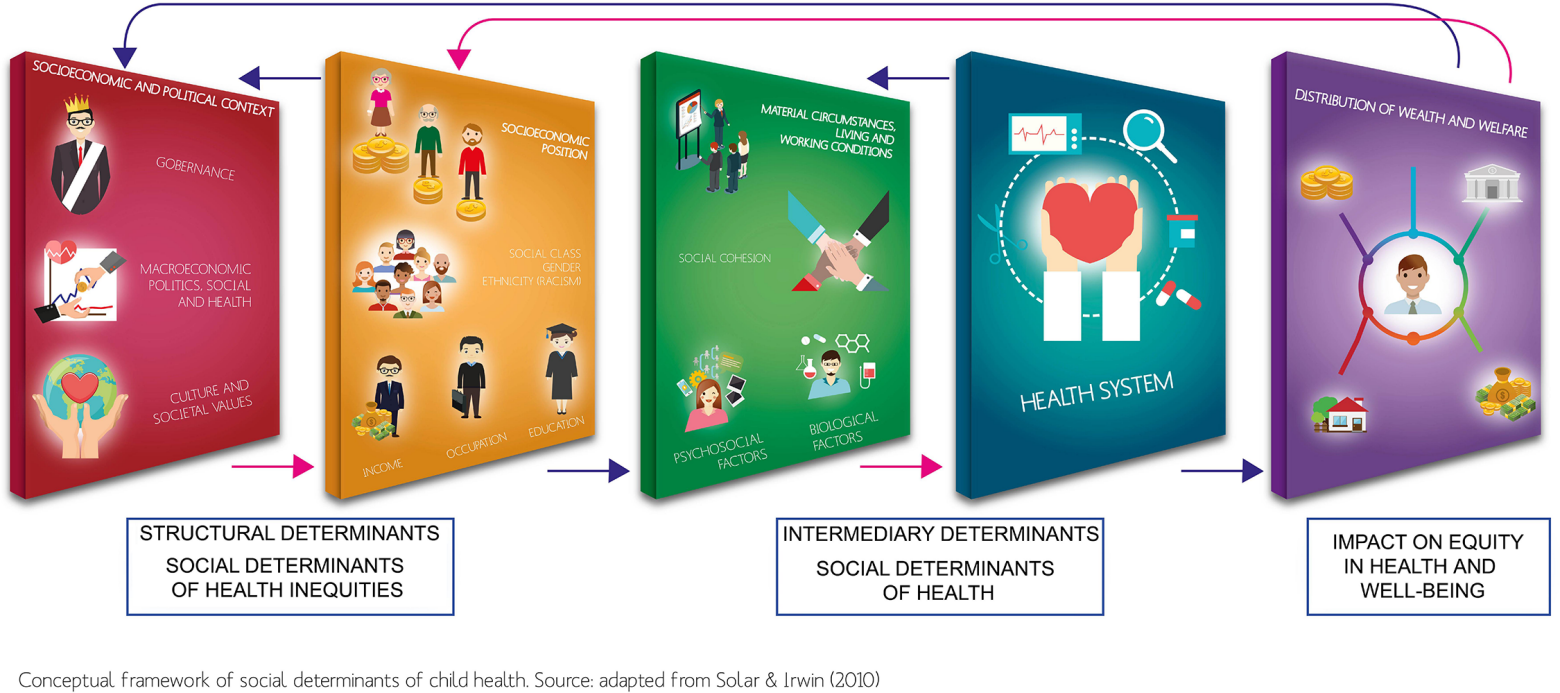
Summary of the World Health Organization’s Social Determinants of Health framework. Interactions of different levels of causation define the structural and intermediary mechanisms, but also the Health System as a social determinant of health plays an very important role in mediating the different outcomes of diseases that generate health inequities.

Historically speaking, worldwide health agendas have been winding their focus either on technologically-driven medical and public health interventions or in understanding population health as a social phenomenon, that can be modified by complex intersectorial policies. The main goal fo the CSDH-FW is on the latter, working under strong commitment to *health equity* and *social justice*. Fig 2 presents a diagrammatic view of the CSDH-FW. From left to right, we can see how the hierarchy of structural and intermediary determinants contribute to shapen the state of health and well-being of the populations. Right-to-left arrows indicate how intersectorial policies may proceed from a population basis, affecting the public health and societal sectors, all the way up to incide on governing agencies and socio-economic governance entities.

The most relevant structural strata also called **structural determinants** (represented by the red and yellow boxes on Fig 2) include social, economic and political context (governance, macroeconomic policy, social policies, public policy, culture and societal values and epidemiological conditions), socioeconomic position, income, education, occupation, social class, gender, race and ethnicity. These underlying social determinants of health inequities operate through a set of **intermediary determinants** of health (green and blue boxes in Fig 2) in shaping health outcomes. The main categories of **intermediary determinants** are; material circumstances, social-environmental or psychosocial circumstances, behavioral factors, biological factors and the health system itself as the mediating the differential consequences of health-illness process in people’s lives [6].

In general, structural determinants are *actionable*, i.e. they can be modified by means of an action or a series of actions e.g. by the effects of public health policy. Intermediary determinants are, on the other hand not subject to outside modifications and often do not change at all.

A first step towards an integrated understanding of how social issues may contribute to determine the cardiovascular health status in human populations, consists, not only in *enlisting* them, but also in sketching the interplay that these features may have among themselves to give rise to the observed impact of social constraints upon population-level health conditions.

Meaningful relationships among the different SDCVD will allow us to have a complete picture of how social phenomena impact cardiovascular health. Such connections, however, are quite difficult to unveil or highlight by resorting to traditional systematic reviews and meta-analyses that usually present the information about the different SDCVD fragmented, or at most, integrated according with the subjective appreciation of the expert reviewer [7, 8]. Other methodological approaches to systematic analyses of the literature have been used recently [9–13], some of them resort to computational mining of the publication databases and archives, semantic networks and ontology-based methods to add context to theme-driven systematic surveys of the literature.

### Aims of the current study

The present study intends to help us understand, at a large scale, multivariate level, how the social determinants of health may influence cardiovascular diseases. What are some of the interrelationships among them and how do they fit into their particular geopolitical and historical contexts. To address these questions, we aim to take advantage of the vast corpus of literature already published in the field, while trying to remain as unbiased as possible in our analyses.

The approach we have decided to take implies resorting to a hybrid scheme involving a computational approach of automated literature mining and discourse analysis techniques supplemented with an ‘a posteriori’ discussion of the main findings.

In order to properly account for these phenomena, it is necessary to start with a consideration of those social determinants of health related to cardiovascular disease already identified –in the current literature– and then search for possible interrelationships among them. In this sense, the main purpose of this work is to perform a screening analysis based on a systematic search of the published scientific information about the SDCVD, and to use this analysis as a starting point to unveil their hidden complexities by resorting to data analytics, complex network analysis and visualization techniques that may allow us to see more clearly the relationship among them in order to better understand the effects of these social determinants on cardiovascular diseases [14].

In consequence, the approach followed in this study was as follows: First, we assembled a preliminary corpus by mining the entire PubMed database for all the papers related to social determinants of cardiovascular disease as denoted by corresponding Medical SubHeading (MeSH) classifiers. Then we performed a curation of the corpus to discard non-relevant content and code the information content, using both manual and automated techniques. Once we had a curated corpus, we built semantic networks (using co-occurrence of MeSH terms as links) and performed topological analyses of such networks to find associations between the different SDCVD. To provide context to such interrelationships, our study includes a detailed analysis of the historical trends. Finally, we discuss the main findings of all these stages aiming to contribute to generate an integrated framework for SDCVD.

## Analysis

The systematic approach may be briefly summarized in a series of study phases or stages, as follows:

**Stage I** In this systematic approach we first analyzed the SDCVD using network theory [15] and scientometrics models formed by systematic key terms [13] –as given by the Medical Sub Heading ontology (MeSH) developed by the National Library of Medicine of the U.S., in particular by the National Center for Biotechnological Information (NCBI) PUBMED database– that describe each SDCVD according to the CSDH-FW.

**Stage II** After that, we searched for relevant studies based on systematic keywords or MeSH terms in the title and abstract. The retrieved literature was reviewed in order to identify duplicated PubMed identifiers (PMID) and exclude them; eligible records information was organized according to the SDCVD criteria using both a manual and a computational approach to discourse analysis.

**Stage III** Heatmap visualization was implemented to depict the main countries and dates from publications.

**Stage IV** Python code was used to design the baseline structure for the network analysis, which was later carried out with Cytoscape, a software platform for network-based analytics, see Fig 1. Information about these methods is further detailed in the corresponding section.

In what follows, we will discuss each one of these research stages to a greater detail.

### Stage I: Literature mining strategy

An initial but crucial step towards a systematic analysis of the literature in a given field consists in developing non-biased approach of information retrieval over comprehensive and *trustworthy* databases. To this end, an automated search of the PubMed / MEDLINE database –which is the largest database of biomedical literature in the world– was conducted. It is indexed by using MeSH terms, which are a collection of selected words or phrases that are able to represent specific biomedical concepts, with a structure dictionary organized as an acyclic graph or tree in which the information tag contents are ordered alphabetically and arrange in a hierarchic fashion. The MeSH dictionary is actually an *ontology*, its structure formalizes the name and definition of entities and their properties in a taxonomy-like manner with interrelationships. The importance of ontologies lies in the compartmentalization of the information, a property that enable the implementation of algorithmic approaches to those entities.

When performing manual or automated searches through the PubMed engine, it automatically spans through all the related MeSH terms to limit the search and hence, making it faster and reliable. This highly structured index database (PubMed/MeSH) is thus an extremely valuable resource for high throughput literature-mining efforts such as the one presented here. In some cases the MeSH classification includes the same term twice, with one of the instances preceded by an asterisk (*). Those entries refer to a MeSH term recognized as a major main topic of an article or a class. For the purposes of this study, such terms were treated as separate entities and analyzed accordingly.

The scientific literature search, was conducted for articles on social determinants of health related to cardiovascular disease indexed in the PubMed/Medline database and published between 1980-2016 (July). PubMed search results were saved into a plain-text Mongodb database document, then a computational literature mining procedure was performed using Python pickles to extract the information into either a corpus document or (as we will see in the upcoming Stage IV subsection) to a network-structured file with the NetworkX Python library.

In brief, the strategy consisted in retrieving the PubMed search (unstructured) and then sorting and storing the information into a structured database that allow for customized query searches and qualitative analysis.

All related source code for general text-processing may be found in https://github.com/CSB-IG/literature/tree/master/text_processing. Specific code for this work is found at https://github.com/CSB-IG/bibliometrics. The codes for the *articles by country* and *articles by yearly queries* can be found there too. The search was not restricted by language, article type, text-availability, PubMed commons, or species. The MongoDb text database for this search is included as S1 Text.

### Stage II: Qualitative analysis of discourse

The last few years have witnessed important improvements in automated data-retrieval processes and natural language processing as applied to information harvesting from large databases. However, it is still necessary to perform manual and computer-assisted assessment of the information recovered using those means. In the present work, the literature corpus generated by automated search in the PubMed database –as outlined in the previous subsection (Stage I)– was subject to a curation process by human inspection and to further computer-aided coding.

Retrieved records were hence submitted to a Qualitative Analysis of Discourse (QAD) (also termed *coding* in the Social Sciences) which is a series of techniques developed to facilitate the interpretation of textual corpus, intended to organize the data by *reading* the corpus and *demarcate* or tag portions of a certain concept or category. There are several approaches for QAD but the most popular are *recursive abstraction*, *coding and thinking* and *hermeneutical classification* [16].

With the advent of computation and natural language processing techniques, it has become possible to implement automated methods for the electronic qualitative analysis of discourse (e-QAD). The purpose of such tools is to provide ways to unveil and analyze hidden patterns in complex and large unstructured databases, particularly those of qualitative nature. These tools allow the researchers to locate, code and annotate their corpus of interest, and also to perform higher level analyses such as evaluation and visualization of trends and concepts.

For this systematic approach we used ATLAS.ti version 0.7 a software for the e-QAD. The first step was to build, name and define categories in the data, according to MeSH terms relate to CSDH-FW and to known cardiovascular risk factors, then analytical units were selected in order to assign them codes and interrelationships. Data sources associated with a specific code were coded and reviewed, by creating *quotations* (tags in the electronic records for the data), and then the entries tagged report were retrieved through the *code manager* tool [17]. A final list of PMID records, driven from CSDH-FW, were generated to identified codes groups (see Fig 2) such as;

- *Political context of health inequities* that included global governance, macroeconomic policies, social policies (as a labour market, housing or land) and any other public policies (education, health and social protection), also culture and social values.
- *Structural determinants of health inequities* such the socioeconomic position and social class (education, occupation and income)
- *Intermediary determinants of health*, material circumstances (living and working conditions, food availability), behaviours, biological and psychological factors as well as health system

It is relevant to highlight that the curatorial procedures followed in this work are based on systematic and (whenever possible) objective criteria, even if it was not a completely computational curation but a hybrid approach.

### Stage III: Data visualization of historical trends

Social phenomena do not occur in isolation of historical and geopolitical circumstances. The same can be said about academic discussion on these issues. One goal of this study is to provide a framework for the analysis and interpretation of the social determinants of cardiovascular disease and their interplay. To contextualize the problem, we looked upon the historical trends in the written academic records for studies in SDCVD by authors from different countries over a span of more than thirty years; since the issue was first discussed in the biomedical literature.

**Fig 3:**
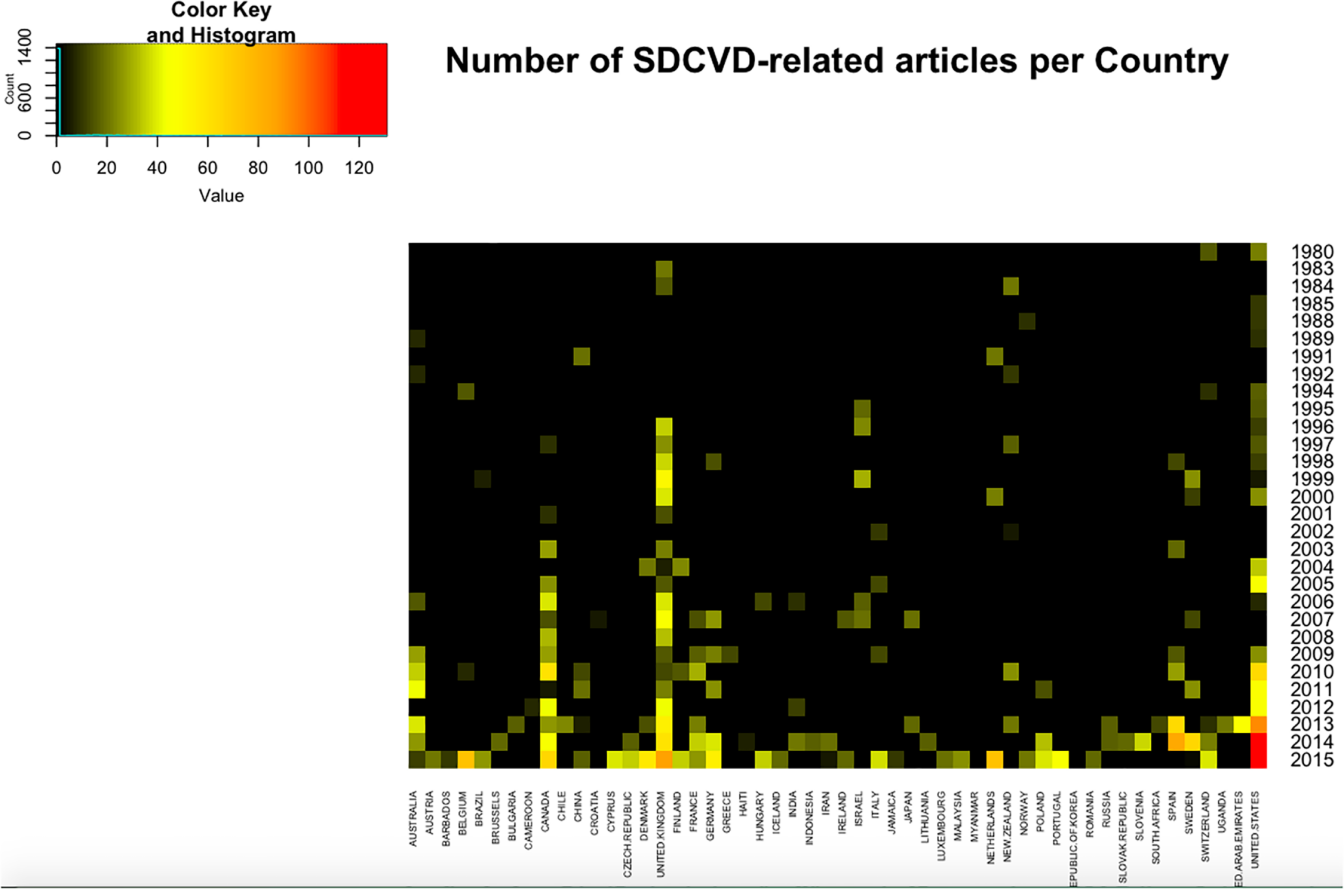
Heatmap visualization of the time evolution of publication activity in the Social Determinants of Health area in different countries. Principal countries are registered by ascription of article in papers related to Social Determinants of Cardiovascular Disease. Bars represent the sum of articles over the period. We can notice that a few countries contributed the vast majority of papers. Number of papers related to Social Determinants of Cardiovascular Disease published by year. Bars represent all papers published in the field within the given year. We can notice a progressive growth on the number of publications in the field over time.

A heatmap visualization (rendered in Fig 3) was built to analyze which nations are more active regarding research in SDCVD. The countries of corresponding authors were placed in columns and the year of publication in rows. The color scale represents the number of times a publication of a certain country appears in a year, then black means there were no publications, while red indicates that a certain country is actively publishing in the field on that given year. Country and year mining were performed with custom-made Python scripts (articles_by_country.py and articles_by_year.py, respectively) available at https://github.com/CSB-IG/bibliometrics. Heatmap visualizations were built by using the *heatmap.2* package included in the *gplots* [R] library.

### Stage IV: Computational methods for network-based discovery

Research methods based on network-based analysis are growing in relevance in recent times. This is so, since they provide with an integrated view that allow the discovery of associations and interactions between the different features characterizing a given system. In the present context, network analysis are intended to unveil hidden (or at least non-evident) interconnections between the different SDCVD, as they have been reported in the comprehensive corpus of current medical literature under examination.

In order to identify the interrelationships among the MeSH terms related to SDCVD, we generated a connectivity map from a curated corpus of biomedical scientific papers published between the years 1980 and 2015 (due to the delay in the NCBI assignment of MeSH term classifiers, no 2016 papers -or newer- were included in this analysis) that were tagged with the systematic approach of the literature. The connectivity maps were built, so that sources and targets nodes are the MeSH terms that identified the papers in corpus and a link between these nodes was drawn if two articles shared additional MeSH terms, the more MeSH terms shared, the stronger the link and hence the closer the connection of these papers was assumed. The IDs in the network construction were the PMID’s of each publication [13]. The computational details of the mining strategy are sketched in the Methods section and described comprehensively at the associated GitHub repository (https://github.com/CSB-IG/bibliometrics).

#### Network topology and analysis

A first step towards the understanding of the web of interrelationships among items connected on a network is the determination of the network’s local and global connectivity patterns. Such *topological features* as the individual and global number of connections (called the *degree*), how are these connections assigned to the different nodes (the *degree distribution*), how important are certain nodes in the networks (called the *centrality measures*), etc. are the ones that will be used to discuss the relative importance and interplay of the different features related to SDCVD.

Once we have a structured corpus, MeSH-network extraction was performed with the Python code included in https://github.com/CSB-IG/bibliometrics/blob/master/mesh_network_from_medline.py, and network analysis with Python’s NetworkX library [15] and Cytoscape version 2.8 [18] with the NetworkAnalyzer plugin [19]. Visualization was performed using Cytoscape [20].

Networks were built with the SDCVD MeSH with which articles were indexed, and the links between the nodes are the PMIDs. Visual parameters used to filter the networks were: connectivity degree, (small sizes for low values) and clustering coefficient (bright colors for low values). Edges are colored by their corresponding value of the edge betweenness statistic (small sizes and bright colors for low values).

**Fig 4:**
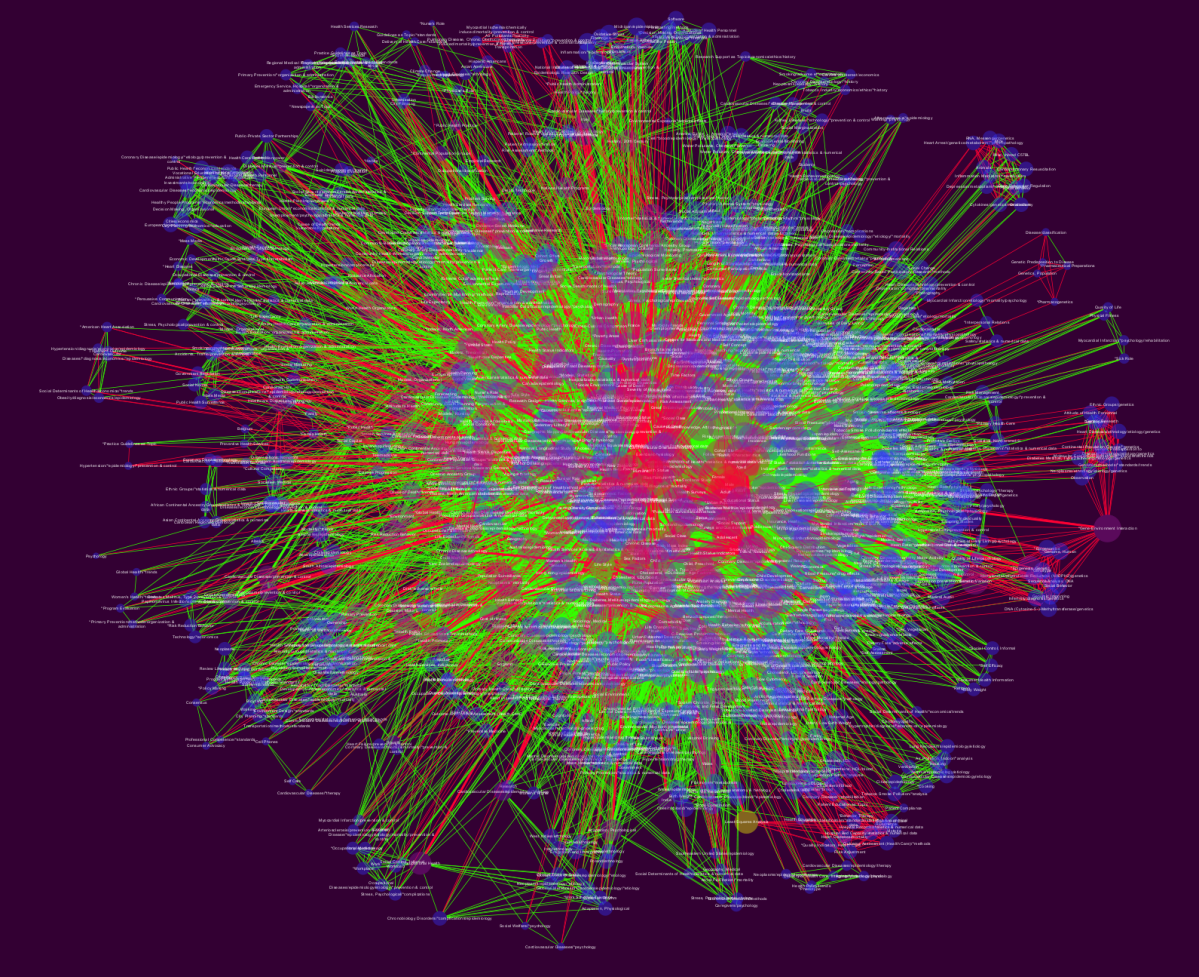
Raw MeSH network for SDCD for the period (1980-2015). This network presents the data structure *before* a manual curation procedure was performed. From this network, once redundant or biased features were removed we obtain the Curated Global Network shown in Fig 5.

The network topology analysis was carried out and we analyzed and report the clustering coefficient, network centralization, and average number of neighbours. The raw (non-curated) network showed in Fig 4, was formed by one connected component with 1,229 nodes and 18,451 edges, the rest of simple parameters are in Table 1.

**Table 1:** Topological features for the analyzed networks. *N*, number of nodes; *M*, number of edges; *CC*, clustering coefficient; *ℓ*, Characteristics path length; *k*, Avg.number of neighbors; *p*, network density.

Network	*N*	*M*	〈*CC*〉	〈*ℓ*〉	Centralization	〈*k*〉	*ρ*
Raw	1229	18451	0.871	1.990	0.960	30.026	0.024
Global	1037	11830	0.880	2.327	0.611	22.816	0.022
SDH	168	1891	0.800	2.071	0.535	22.512	0.135
HSD	211	2750	0.797	1.959	0.610	26.066	0.124
HP	104	869	0.782	2.147	0.448	16.712	0.162
WHO	32	197	0.876	1.603	0.643	12.312	0.397
GH	140	1525	0.804	1.970	0.498	21.786	0.157

For the purpose of this systematic search, those MeSH not related to SDCVD such as; human beings, country, statistical analysis or methods were filtered-out. In order to ensure the comparability among the sub-networks, all of them were filtered by selecting the MeSH with all its nodes and edges, that is, all its first neighbours in an undirected form in which the interaction among these MeSH terms produced a sub-network.

## Results

### Stage I: Literature mining

The search yield 1,190 articles, after abstracts were retrieved and reviewed (title and abstract) 923 were excluded since they did not deal with SDCVD, we assessed the remaining 232 records for eligibility, that were then fully retrieved and reviewed, see Fig 1.

We included only articles that contained at least one MeSH related to the CSDH-FW for cardiovascular disease such as; acute coronary syndrome, myocardial infarction, heart disease and angina pectoris. We excluded articles related to known cardiovascular risk factors (obesity, smoking, physical inactivity, diabetes mellitus, stress, metabolic syndrome, hypertension, cholesterol) or social determinants for different diseases (rheumatic fever, lung diseases, lupus erythematosus, cancer, pneumonia, dementia, Parkinson’s disease).

### Stage II: Qualitative analysis

Then we analysed each title and abstract independently using predefined inclusion criteria based on CSDH suggestions, finally we use a narrative synthesis method by means of ATLAS.ti. After that, we selected the articles for eligibility and classified them by codes according to CSDH-FW. We found 37 articles with full information on SDCVD and articles associated with cardiovascular disease and some code groups of social determinants of health;

Social and economic conditions (51 articles), public policy and health policy (31 articles), health system (18 articles), environment (14 articles), psychological mechanisms (15 articles), geographic location (12 articles), biological mechanisms (11 articles), race and ethnicity (10 articles), education (8 articles), health knowledge and literacy (6 articles), life course or life styles (7 articles), behavioural mechanisms (7 articles). We also found a small number of articles related with adverse childhood (6 articles), employment and occupational status (5 articles), culture and language (5 articles), social support and social networks (4 articles), neighbour or residence characteristics (4 articles), perinatal events (4 articles), vulnerable population (3 articles), marital status (3 articles), health status disparities (2 articles), social environmental (2 articles), income or income inequality (2 articles).

MeSH about Social Determinants of Health (SDH) were identified by means of a hermeneutical method (narrative synthesis) using ATLAS.ti software. This way, we identified highly relevant records about SDCVD as well as the main structural and intermediary determinants according to the CSDH-FW Fig 2. It results noteworthy that even if the literature search and mining strategy was centered around the SDH, there is still an outstanding prevalence of primary risk factors associated with CVD. It is only recently that social determinants of health gained greater importance.

Our findings are consistent with other studies that have shown that from the 1960s on, a growth in the number of studies assessing risk factors for coronary heart disease, that has evidenced two prominent features related with CVD: *the central importance of a small set of risk factors and treatments, and the emergence of social gradients* [21].

### Stage III: Historic data visualization

#### Timeline for publications on SDCVD by country

If we refer to Fig 3, we can see a report of the number of articles from each country by year of publication. In the columns are displayed the main countries (52 of them) that have published about social determinants of health related to cardiovascular disease, rows depict the years of publication from 1980 on. A numeric color code is presented at the upper-left corner, the number of publications grows from black to red.

It is actually possible to discern some temporal patterns or *stages* spanning from around a decade each: the first one ranging from 1980 to around 1990, the second from 1991 to 2000, and so on. We can notice that in the former years there was a low number of publications on the field scattered around very few countries. As time goes by, one may see how the field has been gradually establishing itself in terms of publication rates.

Let us look closer to the historical trends just noticed on a decade by decade basis:

**1980-1989:** During this decade, countries such as the United States, Switzerland, Norway, New Zealand, United Kingdom, China and Australia were the only players in the field.

**1990-1999:** In this period a transition occurs; other players such as Israel, Sweden, Spain, Netherlands, Germany, Canada, Brazil, and Belgium enter the SDCVD arena. During this period, and particularly since 1996, the United Kingdom became a central referent in the field with an almost uninterrupted publication record from that time on. The case of the United States –being the second strongest contributor to the field– is a little bit different: its publication record grows slightly from 1994 to 1998, slowing down in 1999 and regaining force since then at a somehow unsteady pace.

**2000-2009:** It is at the beginning of the 2000’s that Canada starts gaining a relevant place in the field, that will indeed maintain up to this day. Also in this decade, but on the second half Australia establishes as another important player in the study of SDCVD.

**2010-2015:** In recent years, the field was established with many worldwide players, including United Kingdom, United State, Canada, Australia, France, Germany, New Zealand and Spain. Belgium, Brazil, China, Denmark, Finland, Hungary, India, Italy, Japan, Netherlands, Norway, Taiwan and Sweden are also becoming emerging contributors to this field.

### Stage IV: Network analysis

Medical subheading term (MeSH) networks were introduced as a tool to discover conceptual and semantic interrelationships in the SDCVD literature. In Fig 4 we present a network built with the whole (computer-curated) data set. It can be noticed that is a quite **complex network** having a large number of nodes (or *concepts* as given by specific MeSH terms): 1229 and also a large number of links or edges: 18451. Relationships among MeSH terms are given by co-occurrence in one or more research papers. The more papers link two MeSH terms the stronger the interaction as depicted by the width of the link in the network.

A manual curation process revealed that some of the MeSH and relationships included in the so-called *raw* network as obtained by the computational data mining do not correspond to proper instances of SDCVD issues, these were discarded to give rise of what we call a *Global MeSH network*. It is noticeable that we decided to include the raw network in order to follow an open science best-practice to present raw data results and in this way ensure reproducibility on follow-up studies. A rendering of the raw network is included as S1 Fig.

#### Global MeSH network

After a manual curation procedure –back supported by the use of the ATLAS.ti tool– we obtained a network (which we called the Global MeSH network) that is also quite complex, but represents best the true relationships among SDCVD conceptual items as tagged with the corresponding MeSH terms.

**Fig 5:**
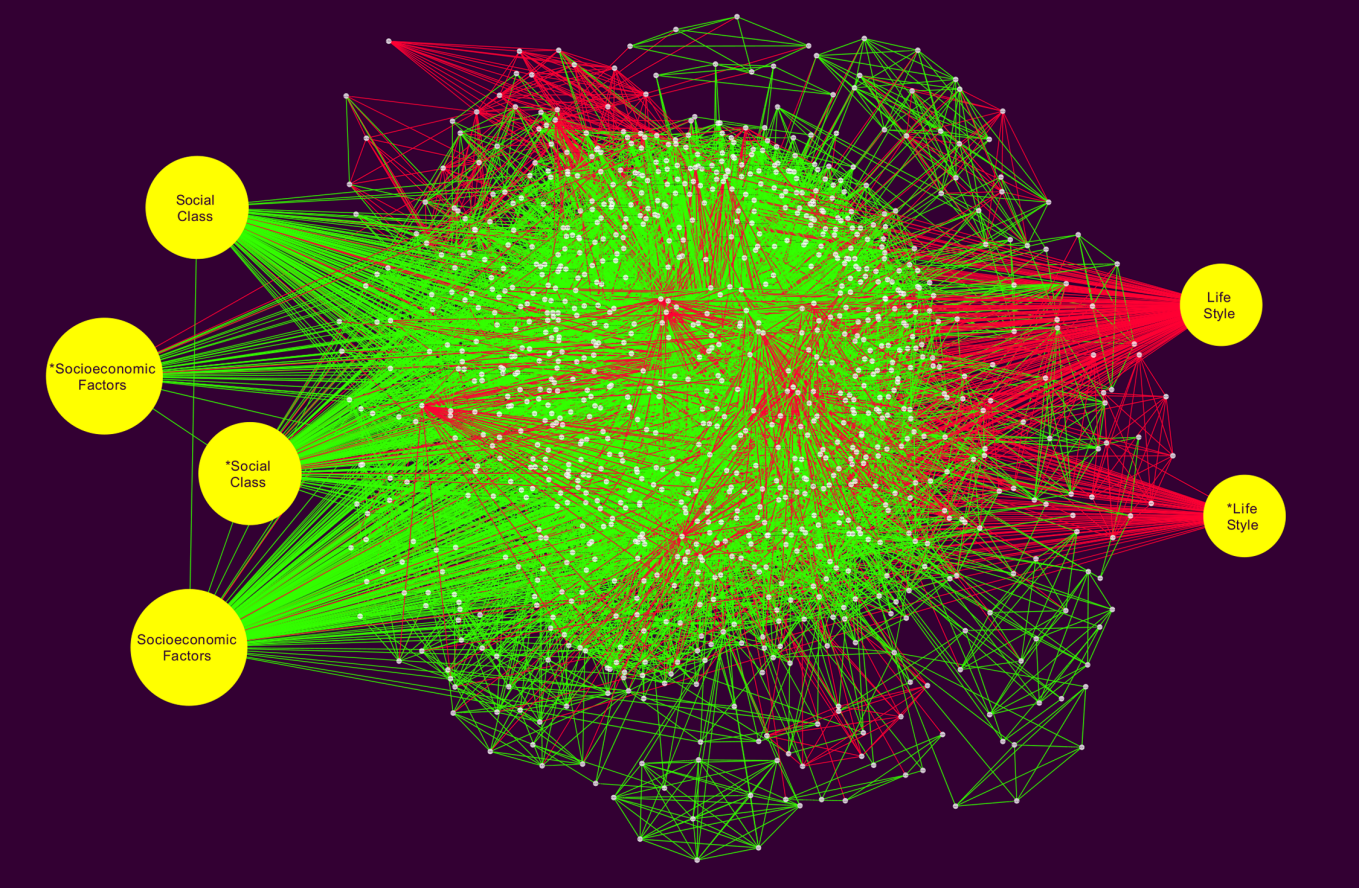
Curated Global MeSH network for SDCD for the period (1980-2015). This network and its subnetworks are the main study object of the MeSH network semantic-like analysis of Social Determinants of Cardiovascular Disease.

**Global MeSH network statistics:** This network is shown in Fig 5 and has one connected component containing 1,037 nodes and 11,830 edges. The global network is indeed a quite large network that, as we will see, it reflects some aspects of complex phenomena related to SDCVD. The mean clustering coefficient of the global network is 0.880 which is large, that aside presents a short characteristic path length of 2.327 (of order log(*N*) with *N* the number of nodes in the network) are indicative of a *small world network* topology. Network centralization is 0.611, average number of neighbors 22.816, and a density of 0.022.

**Global MeSH network main players:** Top 10 highest degree terms are *Female* (degree k = 655), *Male* (k = 625), *Middle Aged* (k = 582), *Risk Factors* (k = 546), *Adult* (k = 532), *Aged* (k = 445), *Socioeconomic Factors* (k = 327), *Social Class* (k = 214) and *Lifestyle* ((k = 172). All of these terms are well-above the average connectivity (i.e. these terms are highly connected nodes or *hubs* in the MeSH network), we see that terms such as *Socioeconomic Factors*, *Social Class* and *Lifestyle* are important social determinants of disease that act as hubs. Later on we will discuss, how these hubs and its connections contribute importantly to shape the MeSH space semantic network on social determinants of cardiovascular disease.

**Global MeSH network relevant categories:** In order to highlight important MeSH terms that are hubs in the network –and will be discussed more thoroughly on the rest of this work– their corresponding nodes appear drawn much larger and yellow colored in Fig 5. These are coded in three main categories: *Socioeconomic factors*, *Social class* and *Lifestyle*. Links are colored in order to distinguish between connections linking *structural determinants of health* (green edges) and those linking mainly *intermediary determinants* or *mediators* (red edges) according to the WHO Framework on Social Determinants of Health [3]. It is worth noticing that the hubs *Socioeconomic factors* and *Social class* are mainly associated with structural determinants (green edges) whereas *Lifestyle* is more connected with *intermediary determinants* and risk factors (red edges).

**Fig 6:**
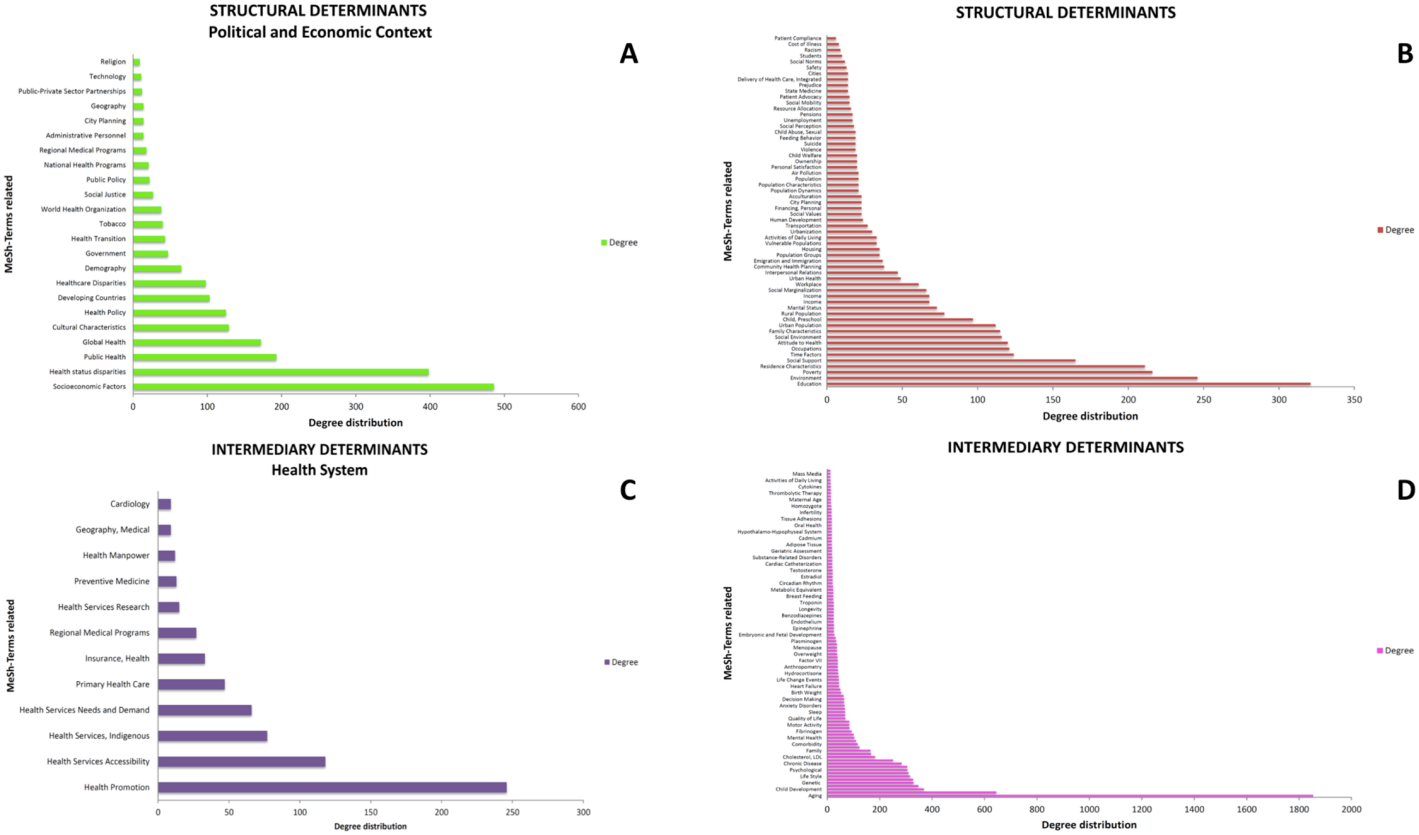
Literature mining results for items categorized according with the WHO’s Social Determinants of health classification (Reference [3]). Histograms represent the connectivity degress for a given MeSH term. Panel **A** presents terms related to Structural Determinants within the Political and Economical Context, Panel **B** presents all other Structural Determinants, Panel **C** shows terms associated with Intermediary Determinants in the context of Health Systems and Panel **D** presents all other Intermediate Determinants.

**Global MeSH network WHO framework:** Since the already mentioned WHO Framework on Social Determinants of Health has become the standard to define policy studies in the field, we decided to analyze to what extent are the items declared on this Framework, present in our Global MeSH network. In order to do so, Fig 6 resumes a coding of the WHO Framework built with the aid of the ATLAS.ti tool. We can appreciate 4 histogram or bar panels, upper panels (A and B) refer to *structural* social determinants. Panel A shows general structural determinants. It is noteworthy that *Education* is the more connected node with a number of links (*k*) of 321. Followed by *Environment* (*k* = 246), *Poverty* (*k* = 216) and *Residence Characteristics* (*k* = 211). Panel B shows MeSH terms associated with *macro-structural* determinants. Here, *Socioeconomic factors* is the more connected term (*k* = 486), followed by *Health Status Disparities* (*k* = 398), *Public Health* (*k* = 193), and *Global Health* (*k* = 172).

Panels C and D refer to determinants coded as *intermediate*. Panel C shows the results for MeSH terms mainly associated with health system factors, that according with our results are in general, amongst the less studied determinants. Here we can see that the more connected term is *Health Promotion* (*k* = 246), followed on a distant second place by *Health Services Accessibility* (*k* = 118), and then we have *Health Services Indigenous* (*k* = 77), and *Health Services Needs and Demand* (*k* = 66). Panel D shows common intermediate determinants and risk factors, here *Aging* is the most connected node (*k* = 1854), followed by *Risk Factors* (*k* = 645), *Child Development* (*k* = 369), *Health Status* (*k* = 348), *Genetic* factors (*k* = 329) and *Smoking* (*k* = 327). A high resolution rendering of the Global network is included as S2 Fig.

### Feature specific subnetworks

Because a main objective of our work is to unveil the role of health inequities, we analyzed the full Global network in the context of the CSDH-FW to build 5 sub-networks based on the following MeSH terms and their first neighbors: *Social Determinants of Health* (SDH), *Health Status Disparities* (HSD), *Health Policy* (HP), *World Health Organization* (WHO) and *Global Health* (GH). On what follows, we will present a detailed account of the results in these subnetworks.

**Fig 7:**
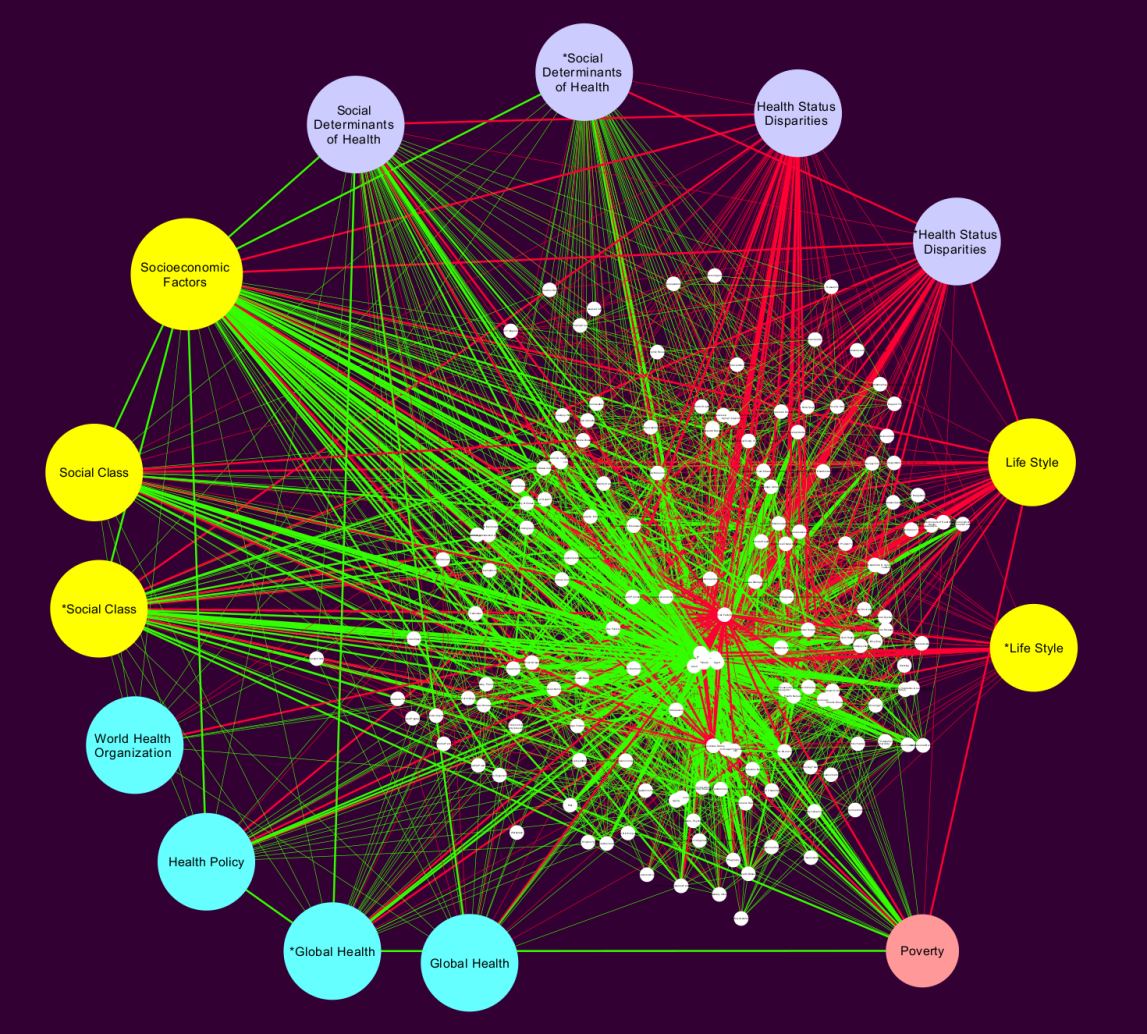
Social Determinants of health subnetwork. This is a subnetwork built from the global network in Fig 5, including only the *Social Determinants of Health* MeSH term and its first neighbors (directly connected terms).

**SDH network:** In Fig 7 we can see the network formed by the *Social Determinants of Health* MeSH and all of its connected first neighbors. This *SDH network* has 168 nodes and 1891 edges. Its mean clustering coefficient is 0.800 is large and a short characteristic path length of 2.071 again suggests a *small world network* topology. Network centralization is 0.536, average number of neighbors 22.51, and a density of 0.135. We were able to see that both networks share the same top degree MeSH terms as those already discussed in the context of the global network.

A set of important terms related to health policy and the way these are related with the social determinants of health arises. These terms are presented as highly connected cyan nodes, including the *Health Policy* MeSH itself, as well as the one related to the *World Health Organization* (that sets the guidelines for health-related global policy making), as well as the MeSH corresponding to *Global Health*, which is one of the most relevant healthcare initiatives in recent times.

A second set of terms that becomes evident in this network refers to the association between social determinants of health and *Health status disparities*, interestingly enough most of the connections of this term are red edges representing an intermediate connection of *Socioeconomic factors* and *Social class* with *Lifestyle*. So, lilac-colored terms present the relationships between *social determinants of health* and *health status disparities*. An interesting term is *Poverty* (here salmon-colored) since it connects social determinants of health with risk factors.

**Fig 8:**
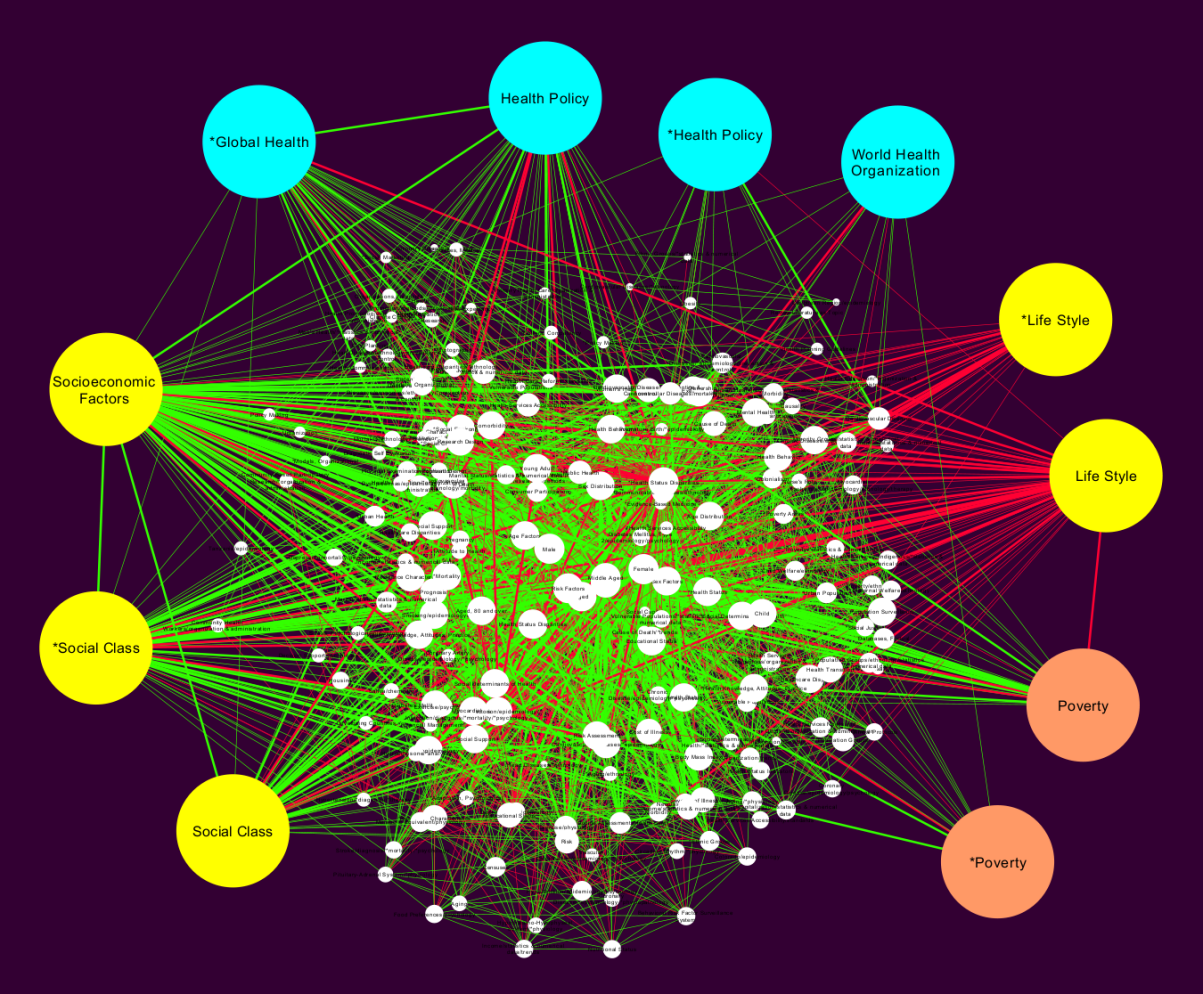
Health status disparities subnetwork. This is a subnetwork built from the global network in Fig 5, including only the *Health Status Disparities* MeSH term and its first neighbors.

**HSD network:** The network related to health status disparities has 211 nodes and 2750 edges, it is shown in Fig 8. Its mean clustering coefficient is 0.797 is also large that aside a short characteristic path length of 1.959 also suggesting a *small world network* topology. Network centralization is 0.610, average number of neighbors 26.066, and a density of 0.124. Health disparities are an associated result of insufficient social programs and policies, unfair economical agreements and faulty political management [22].

Interestingly, in both networks (SDH and HSD), the node corresponding to the MeSH term *Poverty* appears with a somewhat smaller degree (k = 73 in both networks). This fact may indicate that discussion of the role of poverty in the context of health status disparities as a social determinant of cardiovascular disease may be currently still under development.

**Fig 9:**
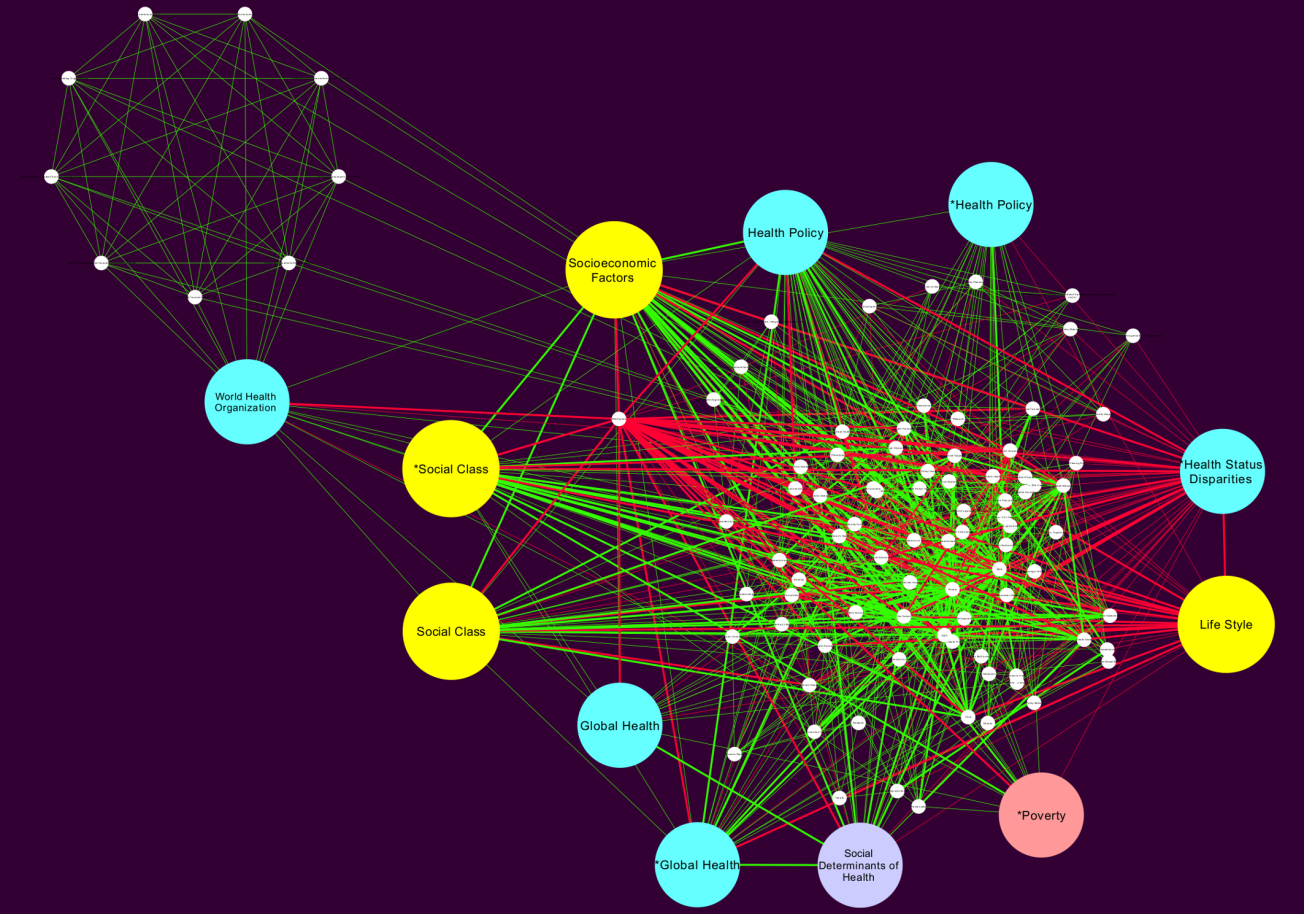
Health policy subnetwork. This is a subnetwork built from the global network in Fig 5, including only the *Health Policy* MeSH term and its first neighbors.

**HP network:** The health policy network has 104 nodes and 869 edges (for further details see S1 Text), it is shown in Fig 9. Its mean clustering coefficient is 0.782, characteristic path length of 2.147. Network centralization is 0.448, average number of neighbors 16.71, and a density of 0.162. Top 10 connected MeSH terms are *Health Policy* (k = 103), *Female* (k = 58), *Male* (k = 56), *Health Status Disparities* (k = 52), *Socio-economic Factors* (k = 51), *Risk Factors* (k = 50), *Adult* (k = 44), *Social Class* (k = 34), *Sex Factor* (k = 34), and *Risk Assessment* (k = 30). We can notice in Fig 9 that unlike in previous networks, the MeSH term *Lifestyle* is somewhat displaced from the hubs (i.e. the 18th highest degree term, k = 26).

Even if this is a smaller, less-connected network, we can see that the main hub terms (colored in yellow, cyan, lilac and salmon as previously described) remain. It is noteworthy that there are a number of terms heavily connected with *World Health Organization* and also among themselves and less connected with the main central cluster. These nodes are the ones representing health policy management, as given by economic decision making and education policy. Apparently, then the WHO it is the natural conduct to large scale link public policy with the social determinants of health.

**Fig 10:**
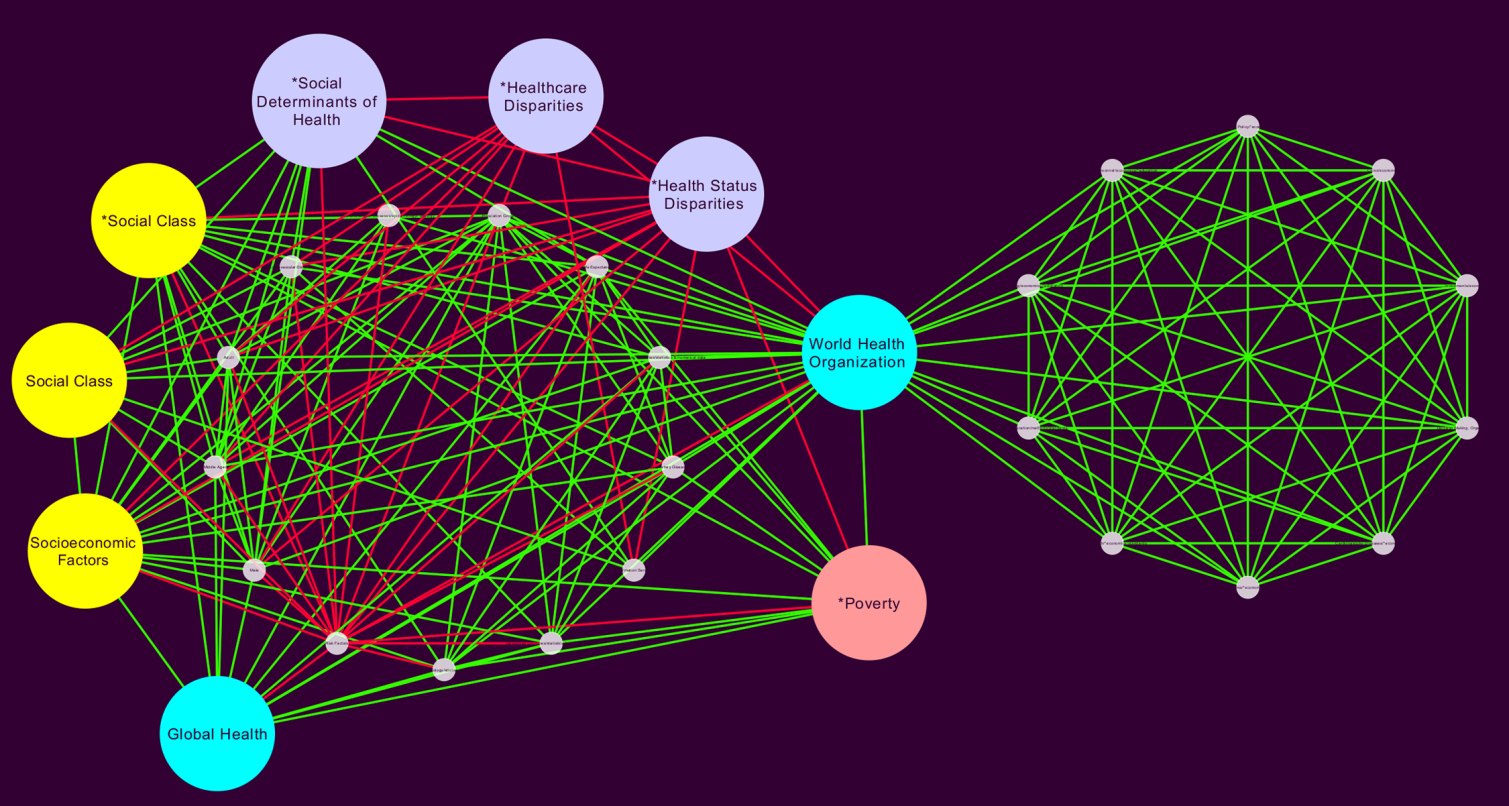
WHO subnetwork. This is a subnetwork built from the global network in Fig 5, including only the *World Health Organization* MeSH term and its first neighbors.

**WHO network:** The WHO-associated network is smaller in size with 32 nodes and 197 edges it is shown in Fig 10. Its mean clustering coefficient is 0.876, characteristic path length of 1.603. Network centralization is 0.643, average number of neighbors 12.31, and a density of 0.397. Top 10 connected MeSH terms are *World Health Organization* (k = 31), *Socio-economic Factors* (k = 19), *Risk Factors* (k = 19), *Population Groups* (k = 16), *Social Class* (k = 16), *Middle Aged* (k = 14), *Male* (k = 14), *Global Health* (k = 14), *Adult* (k = 13), and *Social Determinants of Health* (k = 13). Surprisingly, *Female* and *Poverty* are MeSH terms not found in the network of WHO.

Given the importance that the World Health Organization has shown to possess, we will take a closer look at the network composed by its corresponding MeSH term and related first neighbors as presented in Fig 10. It is also a small network, quite similar to Fig 9, in which terms related to policy management are connected to each other, almost forming a complete graph, that seems to be somehow less connected with the highly relevant hubs such as *Socioeconomic factors*, *Social class*, *Health status disparities*, *Global health* and *Poverty*.

**Fig 11:**
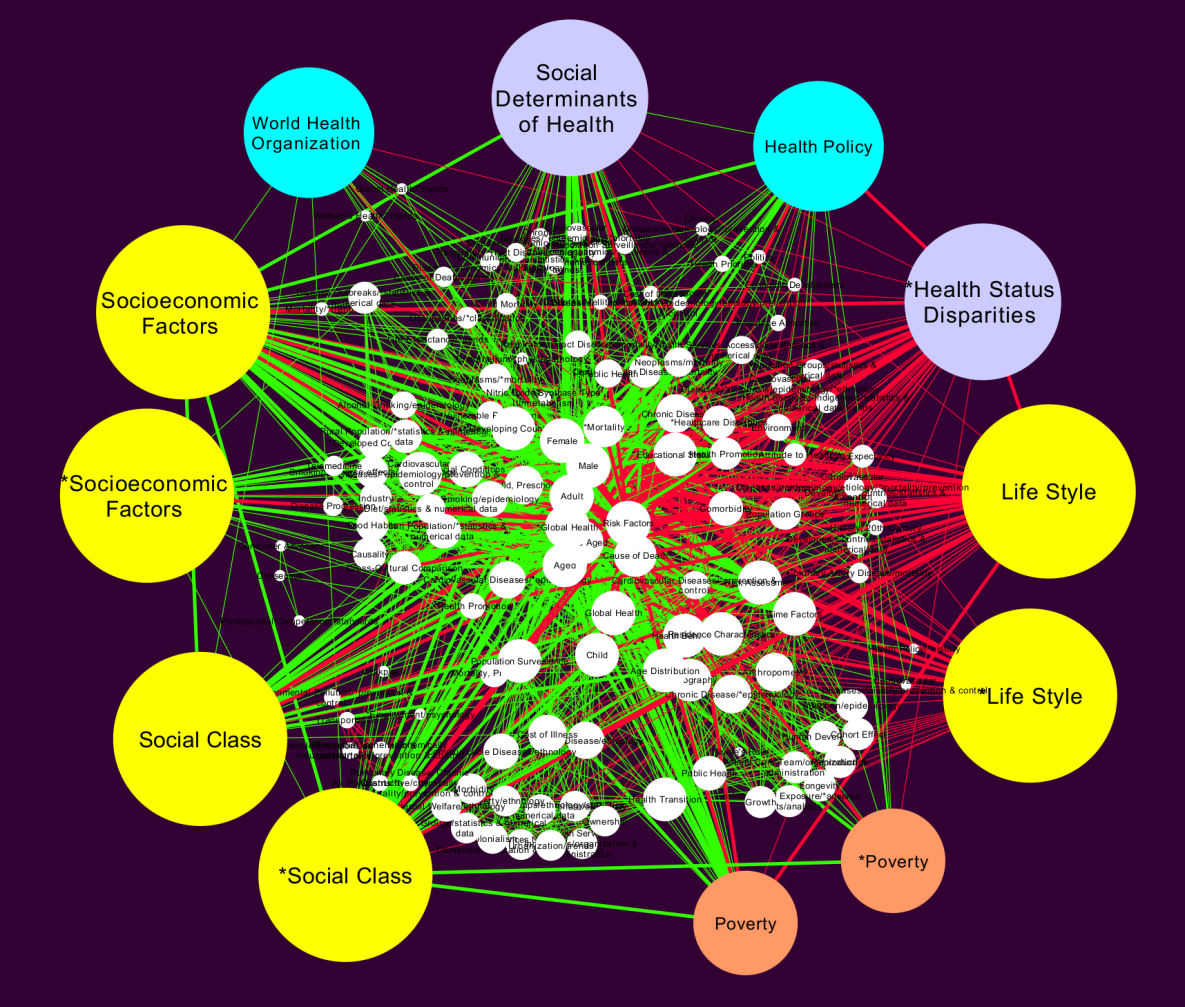
Global health subnetwork. This is a subnetwork built from the global network in Fig 5, including only the *Global Health* MeSH term and its first neighbors.

**GH network:** As already mentioned, *Global health* has been the more ambitious program to outline health policy at the worldwide level and it is a landmark of the influence that the WHO has all abroad. The GH network (which contains *Global health* and all of its first neighbors) has 140 nodes and 1525 edges, Fig 11. Its mean clustering coefficient is 0.804, characteristic path length of 1.970. Network centralization is 0.498, average number of neighbors 21.786, and a density of 0.157. Top 10 connected MeSH terms are *Risk Factors* (k = 90), *Global Health (82), *Male* (k = 78), *Female* (k = 76), *Adult* (k = 67), *Global Health* (65), *Middle Aged* (k = 62), *Socio-economic Factors* (k = 60), *Aged* (k = 57), *Life Style* (k = 55), *Social Class (49).

## Discussion

### Stage I: Literature mining

As a brief note regarding the use of computational approaches to literature mining, we can mention that it presents strong advantages with respect to traditional approaches, since it allows for the consideration of larger, comprehensive corpuses. For the present study, more than a thousand research papers were initially considered. After applying filtering criteria related to the specificity of the issue under consideration, we were allowed to analyze in a deep, systematic fashion more than two hundred of these articles; a task that would have been daunting by resorting to traditional, human-driven approaches.

Computer-aided literature mining allowed us to prepare a comprehensive corpus that sets the basis for structured analyses of a qualitative nature to be performed on a non-biased (or at least, *less-biased*) fashion. Diminishing the effect of personal biases will allow a more systematic and trustworthy qualitative analysis that will help us to improve our discussions on these matters.

### Stage II: Qualitative analysis

The qualitative-analysis strategy taken in this work set the basis for the discussion in the two, separate but interdependent, avenues of research we follow. On the one hand, by using the curated literature corpus as coded using QAD based on the WHO framework, it was possible to study the historical trends present in the academic discussions on SDCVD, as seen on a thirty-plus year period since social determinants of health started to be discussed in the published academic literature.

On the other hand, the same coded corpus was used as the input to build the semantic networks that allowed us to study closely the interplay among the various features and subjects involved in the construction of the SDCVD.

### Stage III: Historic data visualization

As for the historical visualization of published papers, t he term *Social Determinants of Health related to CVD*, was first used in 1980 in the United States of North America and Switzerland, at the same time the Monica Study [23] began with the purpose of monitoring the trends and determinants of CVD.

The first scientific publications on SDCVD took place between 1980 and 1990 in the United Kingdom, Norway, New Zealand, Australia and the United States of North America, probably due to increasing social inequalities that increment the mortality related to CVD [21].

The 1993 World Bank report pointed out the critical role that health investments play in the rising burden of chronic diseases in low and middle-income countries [24], thus triggering the worldwide interest in CVD control and the publication of scientific reports in this decade in Spain, Sweden, Italy, Israel, Germany, Finland, Canada, Netherlands, New Zealand and Brazil, being this last country the first in Latin America (Fig 3).

Between 2003 and 2005, publication production could have been encouraged by three global strategies focusing on the prevention of chronic diseases. 1) The Framework Convention on Tobacco Control [25], 2) the Report on Diet, Nutrition and Chronic Diseases [26, 27] and 3) the Global Strategy on Diet, Physical Activity and Health, all of them revealing that unhealthy behaviours along with non-communicable diseases will be overcrowded among the poorest communities [28]. However, the formalization of the Commission of Social Determinants Health by the WHO in 2005 was definitely the event that impacted more importantly the production of publications on SDCVD from 2005 onwards as Fig 3 shows by the evident increment in light yellow and yellow tiles in the heatmap [29].

Farther parallel phenomena occurring in the world set a trigger for increasing publications related to SDCVD: the report on Preventing Chronic Diseases: Vital investment (2005) [30], the Action Plan for the Global Strategy for the prevention and control of non-communicable diseases (2008) [31], and the first Report from the Commission of Social Determinants of Health in 2008 [29].

All above mentioned events might have increased the awareness of countries already engaged like Ireland, the first country to put into practice the process of plain packaging for tobacco control; followed, by others that started to engage in several strategies (Poland, Japan, Greece, France, Croatia, Cameroon, Australia, Belgium, India and Hungary) (WHO Countries www.who.int/countries/en/). Finally, a country that called our attention was China, that had begun to publish on SDCVD in 1990 but no further publications were found until 2010, even though its CVD mortality rate is 45% may be due to the rapid industrialization, the massive internal migration and the urbanization process which had led China in an enormous social and economic challenge.

The final period, between 2013 and 2015, could be the summary of the implementation of all these strategies which reflect an increase in publications in most countries with diverse socioeconomic situations as Fig 3 shows by the growing in yellow, orange and red tiles in the heatmap.

Finally, Canada, the United States of America and the United Kingdome were the countries with the highest production of publications on SDCVD. The main contributor that influenced the social gradient in CVD was Michelle Marmot who since 1980 has argued that ischemic heart disease has a social crossover in the early stages of the epidemic [32] with an important socioeconomic gradient [33], to our days, especially in connection with the CVD inequalities among the poorest [34–39].

This timeline helped us visualize the Global Health Initiative recently introduce as a strategy to strength the SDH and to understand its multi-and interdisciplinary connections that may improve health conditions through achieving equality among people, which has led to a more complex analysis of the epidemiological transition, the double burden of disease and the inverse social gradient interaction resulting in the decline of CVD in rich countries, while increasing in low and middle income ones or in those where social inequalities are important [40].

### Stage IV: Network analysis

After analyzing the structural (topological) properties of the global semantic network (Fig 5), we notice that is indeed quite large (it has 1,037 nodes and 11,830 edges) and densely connected. This means that there are many issues (more than one thousand), connected to the discussion on SDCVD and that there is a large number of interconnections among them (almost twelve thousand). This implies that, at the semantic level provided by MeSH identifiers, the issue of SDCVD is quite complex. Such a large number of variables and dependencies among them need to be considered closely in further analyses of SDCVD, aimed at the design of public policy and healthcare planning interventions.

When we look at the highly connected concepts in this network, we notice that, the main players in the SDCVD arena are in the one hand, *risk factors* and in the other social determinants of health such as *Socioeconomic factors*, *Social class* and *Life style*. When we distinguish between relationships, between structural determinants and intermediary or mediator determinants according to the WHO framework (as evidenced by edge coloring in Fig 5), we can see that *Socioeconomic factors* and *Social class* are typically connected with structural determinants, whereas *Life style* is mostly connected with intermediary determinants. Such distinguished connections may become relevant on more focused studies, because structural determinants of health are usually non-modifiable, while intermediary determinants can be modified, for instance by life style-adjustment interventions.

A closer analysis of the relative importance of the different social determinants discussed by the WHO framework is given in Fig 6. There we can see histograms depicting the number of links that the principal determinants in each category present in the global MeSH network. Arguably, highly connected determinants for each class, constitute *central* issues that contribute to the integration of the different SDCVD into a single, complex phenomenon.

Regarding the network analysis aimed at looking at health inequities, by following the WHO’s CSDH-FW, five subnetworks were built around *Social Determinants of Health*, *Health Status Disparities*, *Health Policy*, *World Health Organization* and *Global Health*. Such subgraphs may be interpreted as presenting a different *context* with a more specific focus on a given feature. While global, large scale networks are useful to size the scope and limits of the studies on SDCVD as a whole, these feature specific subnetworks will allow us to look upon specific connections of interest. This is so since the number of features goes now in the few hundreds and the number of connections is actually downsized to be up to around two thousand, at most.

As it can be seen in the results section, the study of these subnetworks, help to identify for instance, how *poverty* seems to establish a link between social determinants of health and risk factors (as observed by analyzing the SDH network, Fig 7). A similar analysis of the WHO network in Fig 10, unveil that even though most terms related to policy management are highly connected to each other (implying that the published studies on the matter constitute an integrated body of knowledge), they are, not so well connected with the most important social determinants of health such as socioeconomic factors, social class and health status disparities. This last statement seems to point out to a lack of coherence between the discussions of health policy and the social determinants of health.

The last paragraph provides us with just two examples of how the semantic network analysis performed in this work, can be used to provide context for specific studies on SDCVD and to enhance the scope of the discussion on this highly relevant problem. To this end, we are providing access to all tables and computer-readable files for the networks studied here, that will hopefully be serving as a starting point for future discussions on SDCVD.

### Limitations of the present approach

Although we are convinced of the benefits of the approach we just took, there is no study free of limitations. For instance, our whole analysis was made under the framework outlined by the World Health Organization. Even if this is a quite comprehensive framework, it is not devoid of shortcomings. We are also obviously restrained by the use of the MeSH term classifiers. As already mentioned, MeSH terms conform a highly structured ontology, useful for automated text classification. However, specific concepts, relevant to social determinants of health may not be appropriately rendered to a particular MeSH term. Therefore, we may be losing some specificity in our description.

Since our Knowledge Database approach was bound to literature published and indexed in the PubMed/Medline database, there are a number of publication biases introduced. One of them is the one caused by over-representation of papers from the top publishing countries on the subject. Many of them are actually developed countries for which the overall landscape of social determinants of health may differ from that of underdeveloped economies. Another bias is, of course, positive result over-emphasis, common to most scholarly articles.

In spite of the broad scope of the PubMed/Medline coverage, there is a substantial corpus of information published in journals and books not indexed on it, nor classified by means of MeSH terms. The information in these other sources is not considered in the present study.

Last but not least, there are also methodological constraints related to the use of network analysis. Useful as it is, network analysis provides us with a ‘coarse-grained’ view that highlights large scale relation,s but may mask specific links that may be relevant. Also, a novel approach like this may not be easily generalizable to other cases.

## Conclusion

The enormous rise on the incidence of cardiovascular diseases at a global level (that are becoming tantamount to a world proportion epidemic) calls for immediate action from the public health agencies and international health organizations. Aside from the physiological and genetic basis, it has been widely shown that CVDs possess a highly relevant component of social determinants and risk factors on their ethyology, that become even more important for the design of prevention, treatment, and control strategies [41]. Although SDCVD have been recognized as an important area of research for some time, there is still a need to integrate the vast corpus of literature on the field to recognize some of the complexities that arise from the interplay of the different factors involved in the social, economical and environmental determinants of cardiovascular disease, with particular emphasis on setting guidelines for policy making.

The goal of this study was to use some methodologies of automated literature mining, complex network modeling and computer-aided qualitative analysis of discourse to disentangle some of the intrincacies of the social determinants of cardiovascular diseases and start a discussion not only of the conforming elements under consideration but also on their interconnections and relationships, to move on to try to elaborate models useful for policy making. Rather than performing a classic *meta-analysis* of the literature that, while in principle is more a more straighforward approach, it would be extremely biased toward the quantitative aspects and too *unidimensional* –failing to capture some non-trivial connections that our current study is able to develop, albeit still at a preliminary level.

Unveiling such connections resulted important for us since efforts to change health policy and practice need to recognize that these are complex and dynamic process, whose interactions are shaped by social, political, economic, and organisational factors. There is thus a need to understand the way in which the characteristics of the target population, health professionals, and organizations impact on the effectiveness of health policy. This cannot be done without a proper analysis of the social aspects determining population health and disease [42]. There is of course a long way before all the pieces in the social determinants of cardiovascular disease puzzle are solved, but we are confident that by looking at all the pieces together (by searching the entire literature on the subject matter –as indexed in databases such as PubMed–), by using computer-aided analytics at the semantic (MeSH term networks) and conceptual (qualitative analysis of discourse) levels and by integrating all of these different information layers, we will be able to step forward in the right direction.

## Supporting information

S1 Text PubMed search file. PubMed search MongoDb text file. A database file containing all bibliographical records retrieved by the Systematic Search using as main parameter the *Social Determinants of Health* MeSH term and as modifiers the cardiovascular disease main terms as discussed in the *Literature mining strategy* subsection.
(TXT)

S1 Fig Raw network. PDF file containing the raw network. Such network was generated by the semantic-like associations as given by MeSH term co-occurrence [13].
(PDF)

S2 Fig Global network. PDF file containing the Global network. The Global network was generated by the semantic-like associations as given by MeSH term co-occurrence [13], afterwards it was curated to eliminate redundant and biased terms.
(PDF)
