# Supplementary figures and images for: A systematic approach to analyze the social determinants of cardiovascular disease
Source: PLoS One. 2018 Jan 25;13(1):e0190960. doi: 10.1371/journal.pone.0190960 (PMC5784921; doi:10.1371/journal.pone.0190960)

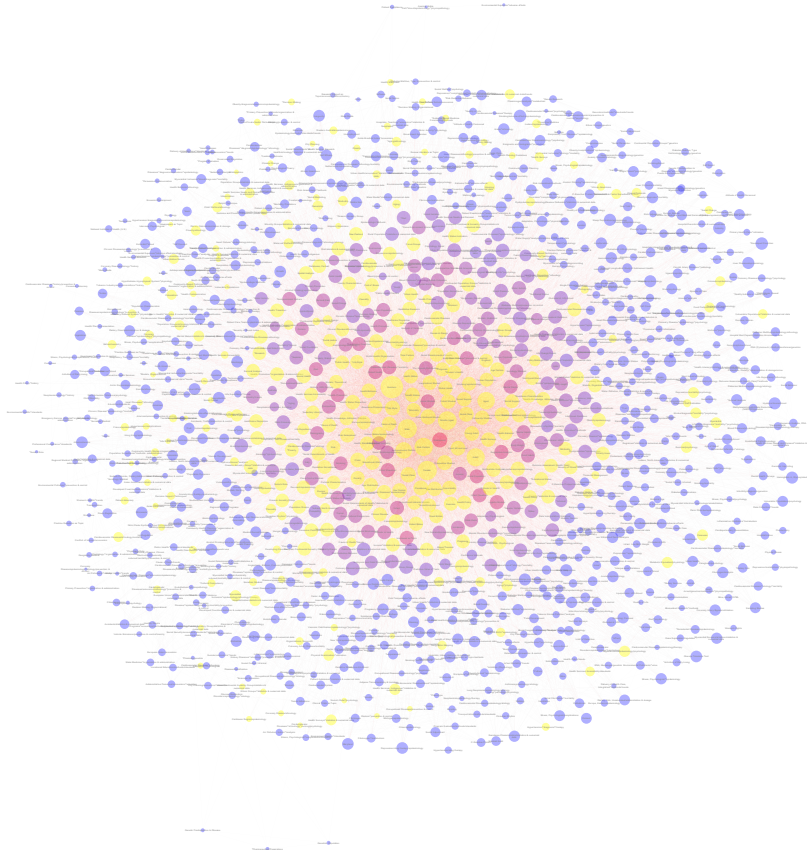

Supplement: S1 Fig — PDF file containing the raw network. Such network was generated by the semantic-like associations as given by MeSH term co-occurrence [13]. (PDF) [file pone.0190960.s002.pdf]

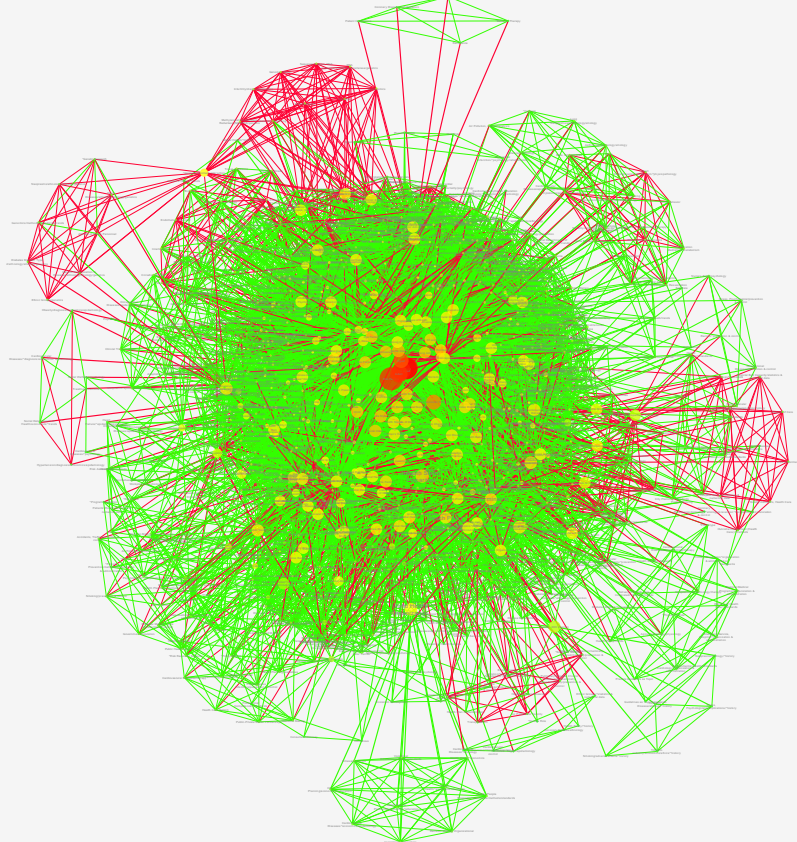

Supplement: S2 Fig — PDF file containing the Global network. The Global network was generated by the semantic-like associations as given by MeSH term co-occurrence [13], afterwards it was curated to eliminate redundant and biased terms. (PDF) [file pone.0190960.s003.pdf]
